# Conserved *cis*-regulatory modules in promoters of genes encoding wheat high-molecular-weight glutenin subunits

**DOI:** 10.3389/fpls.2014.00621

**Published:** 2014-11-12

**Authors:** Catherine Ravel, Samuel Fiquet, Julie Boudet, Mireille Dardevet, Jonathan Vincent, Marielle Merlino, Robin Michard, Pierre Martre

**Affiliations:** ^1^Institut National de la Recherche Agronomique, UMR1095, Genetics, Diversity and Ecophysiology of CerealsClermont-Ferrand, France; ^2^UMR1095, Genetics, Diversity and Ecophysiology of Cereals, Department of Biology, Blaise Pascal UniversityAubière, France

**Keywords:** *cis*-elements, conserved *cis*-regulatory modules (CCRMs), high-molecular-weight glutenin subunits (HMW-GS), transcriptional regulation, seed storage proteins (SSPs), transcription factors (TFs), wheat (*Triticum aestivum* L)

## Abstract

The concentration and composition of the gliadin and glutenin seed storage proteins (SSPs) in wheat flour are the most important determinants of its end-use value. In cereals, the synthesis of SSPs is predominantly regulated at the transcriptional level by a complex network involving at least five *cis*-elements in gene promoters. The high-molecular-weight glutenin subunits (HMW-GS) are encoded by two tightly linked genes located on the long arms of group 1 chromosomes. Here, we sequenced and annotated the HMW-GS gene promoters of 22 electrophoretic wheat alleles to identify putative *cis-regulatory* motifs. We focused on 24 motifs known to be involved in SSP gene regulation. Most of them were identified in at least one HMW-GS gene promoter sequence. A common regulatory framework was observed in all the HMW-GS gene promoters, as they shared conserved *cis*-regulatory modules (CCRMs) including all the five motifs known to regulate the transcription of SSP genes. This common regulatory framework comprises a composite box made of the GATA motifs and GCN4-like Motifs (GLMs) and was shown to be functional as the GLMs are able to bind a bZIP transcriptional factor SPA (Storage Protein Activator). In addition to this regulatory framework, each HMW-GS gene promoter had additional motifs organized differently. The promoters of most highly expressed x-type HMW-GS genes contain an additional box predicted to bind R2R3-MYB transcriptional factors. However, the differences in annotation between promoter alleles could not be related to their level of expression. In summary, we identified a common modular organization of HMW-GS gene promoters but the lack of correlation between the *cis-motifs* of each HMW-GS gene promoter and their level of expression suggests that other *cis*-elements or other mechanisms regulate HMW-GS gene expression.

## Introduction

Wheat is one of the three most economically important crops in the world with maize and rice, with a global annual production of about 700 Mt in 2012 (FAOSTAT; http://faostat.fao.org/). Wheat is a broad term for crops including tetraploid species (2*n* = 28) like durum wheat (*Triticum turgidum* spp. *durum*) and hexaploid species (2*n* = 42) like bread wheat (*T. aestivum* spp. *aestivum*). Wheat is one of the most important sources of carbohydrates and vegetable proteins in human diets as it accounts for about 20% of all calories and proteins consumed. It is mostly transformed before it is consumed, and each type of transformation depends on the unique visco-elastic properties of gluten, a network formed by water and seed storage proteins (SSPs). It is mainly the SSPs that determine the technological quality of wheat flour (for instance, see reviews by Shewry et al., 2002 and Shewry, 2009). Prolamins, the major component of wheat SSPs, comprise monomeric gliadins and polymeric glutenins. The latters have both low- (LMW-GS) and high- (HMW-GS) molecular-weight subunits. Glutenins account for 30–50% of the total SSP content of grain, with HMW-GS alone representing up to 12% of the total. Glutenins strongly influence dough elasticity (Payne et al., 1987; Shewry et al., 2002), with HMW-GS more so than LMW-GS (Branlard and Dardevet, 1985; Gupta and MacRitchie, 1994; He et al., 2005).

As glutenins are so important for technological quality, the genes coding for HMW-GS have been extensively studied. The genome of the hexaploid bread wheat is divided into three sub-genomes (called A, B, and D) forming three homoeologous groups. HMW-GS are encoded by the three loci *Glu-A1*, -*B1* and -*D1* located on the long arms of the group 1 chromosomes. As confirmed by the sequencing of these three regions (Gu et al., 2006), each locus consists of two closely linked paralogous genes, *Glu-1-1* and *Glu-1-2*, that encode x-type and y-type HMW-GS, respectively. Thus, bread wheat HMW-GS form a small multigene family of six genes with two orthologous sets of *Glu-1-1* and *Glu-1-2* genes (Allaby et al., 1999). HMW-GS genes are highly polymorphic (e.g., Payne and Lawrence, 1983). These six genes are not always all expressed. *Glu-A1-2* is silent so from three to five HMW-GS genes are usually expressed in grain. A duplication of *Glu-B1-1* is observed in lines with the overexpressed Bx7 HMW-GS giving an additional expressed gene (Ragupathy et al., 2008). SSPs are specifically expressed in the endosperm and all HMW-GS have similar patterns of expression and represent 60–65% of the total RNA from the endosperm between 10 and 30 days post anthesis (Shewry et al., 2009).

SSP synthesis is primarily controlled both spatially and temporally at the transcriptional level. Transcription factors (TFs) bind specifically to short conserved DNA sequences (5–15 nucleotides) called *cis*-regulatory elements or *cis*-elements, which are usually located in the proximal promoter of genes and characterized by a consensus motif. In barley (*Hordeum vulgare*), the regulatory mechanisms of SSP genes have been extensively studied by transient expression experiments using an hordein promoter (Mena et al., 1998; Vicente-Carbajosa et al., 1998; Oñate et al., 1999; Diaz et al., 2002, 2005; Isabel-La Moneda et al., 2003; Rubio-Somoza et al., 2006a,b; Moreno-Risueno et al., 2008) and have been described as a network of *cis-*elements and their interacting TFs (Rubio-Somoza et al., 2006a). This network is conserved in other cereals as reviewed by Verdier and Thompson (2008) and Xi and Zheng (2011). It consists of five *cis*-elements able to recognize eight TFs belonging to four families (bZIP of the Opaque-2 family, and the B3, DOF, and MYB proteins), which are all reported to be activators of SSP genes. More precisely, the GCN4 like-motif (GLM, 5′-ATGAG/CTCAT-3′) and the prolamin box (P-box, or PB, 5′-TGTAAAG-3′), also called the endosperm motif, constitute the bipartite endosperm box, which plays a key role in activating the expression of prolamin genes as also shown in wheat (Hammond-Kosack et al., 1993). GLM is recognized by bZIP TFs, like BLZ1 and BLZ2 in barley (Vicente-Carbajosa et al., 1998; Oñate et al., 1999) or SPA (Storage Protein Activator) in wheat (Albani et al., 1997), while the P-box is bound by PBF and SAD, both DOF-type TFs (Vicente-Carbajosa et al., 1997; Mena et al., 1998; Diaz et al., 2005). Two additional *cis*-elements, 5′-AACA/TA-3′ and 5′-TATC/GATA-3′ core sequences, are able to bind R2R3-MYB (notably GAMYB) and R1MYB (MCB1 and MYBS3) TFs, respectively (Diaz et al., 2002; Rubio-Somoza et al., 2006a,b). The last *cis*-regulatory sequence is the RY repeat (5′-CATGCATG-3′), which binds FUSCA3, a B3 protein (Bäumlein et al., 1992; Moreno-Risueno et al., 2008). In addition to these DNA-protein interactions, protein-protein interactions consolidate the formation of larger complexes that regulate SSP expression (Rubio-Somoza et al., 2006b).

Wheat promoters of α-gliadin classes (Van Herpen et al., 2008), LMW-GS (Hammond-Kosack et al., 1993; Conlan et al., 1999), and HMW-GS (Norre et al., 2002) have been functionally analyzed. Van Herpen et al. (2008) reported differences in regulatory-elements between promoter sequences of α-gliadin genes from A and B genomes. The LMW-GS promoter studied is characterized by a tandem repeat of two endosperm motifs known as the long endosperm box that is important for controlling endosperm-specific expression (Hammond-Kosack et al., 1993). Thomas and Flavell (1990) and Norre et al. (2002) analyzed extensively the promoters of *Glu-D1* by transient expression assay in tobacco and maize. A 38-bp enhancer element has been identified (Thomas and Flavell, 1990). In addition, the promoter of *Glu-D1-1* contains an atypical endosperm box where the P-box is associated with a G-like box of the ACGT family able to bind bZIP proteins (Norre et al., 2002). Moreover, these authors suggested that the enhancer element may act with the G-like box to increase reporter gene expression.

The exponential growth of genomic sequence databases, and the development of specialized databases of *cis*-acting elements in plants (Higo et al., 1999; Rombauts et al., 1999), coupled with the development of bioinformatics tools to discover specific motifs in DNA or protein sequences (e.g., MEME; Bailey et al., 2006), greatly facilitate the *in silico* analysis of promoters. However, the discovery of *cis*-regulatory elements is hindered by the variability within their sequences, which typically tolerate nucleotide substitutions without a loss of functionality. There are ways of taking this variability into account when predicting the presence of *cis*-regulatory elements (Stormo, 2000). Another aspect to consider is that, in higher eukaryotes, TFs often regulate gene expression by binding DNA in cooperation with other regulatory proteins. As reviewed by Armone and Davidson (1997), separate *cis*-elements of a given promoter often interact with different parts of an overall regulatory complex. This type of organization of *cis*-elements in a region of up to a few 100 bases in the vicinity of the gene being regulated is called a *cis*-regulatory module (CRM), where the relative positions of *cis*-elements and the distances between them are crucial.

Recently, the LMW-GS and HMW-GS gene promoters have been analyzed *in silico* (Juhász et al., 2011; Makai et al., 2013). The *cis*-acting elements present in published sequences of LMW-GS genes, mainly ESTs, were computationally retrieved and differences in the numbers and combinations of specific sequences were highlighted allowing the identification of conserved non-coding sequence regions (CRMs). Models for the transcriptional regulation of LMW-GS genes were then proposed (Juhász et al., 2011). The promoter profiles of HMW-GS genes are highly conserved in the Triticeae family despite differences between paralogous genes (Makai et al., 2013). Here the aim was to understand in more detail the transcriptional regulation of HMW-GS genes through a comparative promoter analysis. The promoters of the main alleles at each HMW-GS gene were analyzed *in silico* for the predicted presence of *cis*-regulatory elements. The organization of these elements within orthologous (homoeologous) and paralogous copies was compared. This work shows the presence of conserved CRMs (CCRMs). In addition, the HMW-GS gene promoters were sequenced in a set of wheat lines to determine whether their sequence variability correlates with the organization of *cis*-elements and hence the expression levels of these genes. A functional analysis of conserved regions consisting of *cis*-motifs potentially able to bind bZIP TFs was carried out by using transient expression and electrophoretic mobility shift assays (EMSA).

## Materials and methods

### Diversity analysis

Forty-two lines representative of the genetic diversity (Haseneyer et al., 2008; Ravel et al., 2009) and of the main electrophoretic alleles of HMW-GS of the INRA worldwide hexaploid wheat (*Triticum aestivum* L.) core collection (Balfourier et al., 2007) were analyzed (Table 1). Genomic DNA was extracted from leaves as described in Ravel et al. (2009) and used for PCR amplification of the proximal promoter of HMW-GS genes. Fragments of approximately 700–1100 nucleotides were obtained (Supplementary Table 1) and sequenced. We did not amplify *Glu-A1-2* genomic DNA as it was silent in all the 42 lines. Diversity indices including nucleotide diversity (π), number of segregating sites (θ), number of haplotypes (H), haplotype diversity (Hd), and Tajima's *D*-test of neutral evolution were calculated for each sequence with SNiPlay (Dereeper et al., 2011).

**Table 1:** **Country of origin, protein coding alleles, and haplotypes of the promoters of five HMW-GS genes for 42 accessions of the INRA worldwide hexaploid wheat core collection**.

**Name^a^**	**Country of origin^b^**	***Glu-A1-1***		***Glu-B1-1***		***Glu-B1-2***		***Glu-D1-1***		***Glu-D1-2***	
		**Protein^c^**	**Promoter^d^**	**Protein^c^**	**Promoter^d^**	**Protein^c^**	**Promoter^d^**	**Protein^c^**	**Promoter^d^**	**Protein^c^**	**Promoter^d^**
A4 (748)	AFG	1	h1	7	h1	8	h1	3	h1	12	h1
Aifeng NO 4 (822)	CHN	1	h1	7	h1	HZ	h1	HZ	h2	HZ	h1
**ARCHE (964)**	FRA	null	h2	6	h2	8	h2	2	h2	12	h1
AURORE (1110)	AUS	2^*^	h3	7	h1	9	h1	2	h2	12	h1
BALKAN (1192)	YUG	2^*^	h3	7	h1	9	h1	5	h3	10	h2
BARBU DU FINISTERE (1323)	FRA	null	h2	20	h1	20	h3	2	h2	12	h1
**BELLIEI 590 (1288)**	HUN	2^*^	h3	7	h1	9	h1	5	h3	10	h2
**CHINESE SPRING (2135)**	CHN	null	h2	7	h1	8	h1	2	h2	12	h1
CHORTANDINKA (2153)	RUS	null	h1	HZ	h1	HZ	h1	HZ	h3	HZ	h2
CHYAMTANG (2171)	NPL	null	h2	7	h1	8	h1	2	h2	12	h1
COPPADRA (2330)	TUR	2^*^	h3	7	h1	8	h1	3	h2	12	h1
COTIPORA (2353)	BRA	2^*^	h3	N	h1	N	h3	2	h2	12	h1
**COURTOT (2358)**	FRA	2^*^	h3	7	h1	8	h1	2	h2	12	h1
DI7202-103 (2526)	FRA	1	h1	7	h1	8	h1	5	h3	10	h2
**GLENLEA (3358)**	CAN	2^*^	h3	7OE	h1	8	h1	5	h3	10	h2
GODOLLOI 15 (3366)	HUN	null	h2	N	h1	N	h4	5	h3	10	h2
JO3045 (3942)	FIN	2^*^	h3	7	h1	9	ND	2	h2	12	h1
M708//G25/N163 (4482)	ISR	2^*^	h4	HZ	h1	HZ	h1	2	h2	12	h1
MARS DE SUEDE ROUGE BARBU (4645)	FRA	null	h2	7	h1	8	h1	2	h2	12	h1
**MISKAAGANI (4874)**	LBN	2^*^	h3	N	h1	N	h1	2	h2	12	h1
MOCHO DE ESPIGA BRANCA (4901)	PRT	2^*^	h3	13	h3	16	h1	2	h2	12	h1
N46 (5088)	ISR	null	h2	7	h1	8	h1	2	h2	12	h1
NABU EPI BLANC (5102)	NPL	null	h2	7	h1	8	h1	2	h2	12	ND
NANKING 25 (5116)	CHN	null	h2	7	h1	8	h1	2	h2	12	h1
NEPAL 84 (5166)	NPL	null	h2	7	h1	8	h1	2	h2	12	h1
NP120 (5308)	IND	null	h2	17	h1	18	h1	2	h2	12	h1
NYU BAY (5399)	JPN	null	h2	7	h1	8	h4	2	h2	12	h1
OPAL (5486)	DEU	1	h1	7	h1	9	h1	5	h3	10	h2
PITIC 62 (5748)	MEX	1	h1	7	h1	8	h1	2	h2	12	h1
**RECITAL (6027)**	FRA	2^*^	h3	6	h2	8	h2	5	h3	10	h2
**RENAN (6086)**	FRA	2^*^	h3	7	h1	8	h1	5	h3	10	h2
**SEU SEUN 27 (6529)**	KOR	null	h2	7	h4	8	h1	4	h2	12	h1
**RALET (8048)**	FRA	null	h5	20	h1	20	h3	2	h2	12	h1
**ZANDA (8058)**	BEL	1	h2	20	h1	20	h3	2	h2	12	h1
**HOPEA (9048)**	FIN	1	N	6	h2	8	h2	2	h2	12	h1
FRUH-WEIZEN (13310)	DEU	null	h2	22	h5	22	h5	5	h3	10	h2
ORNICAR (13471)	FRA	2^*^	h3	6	h2	8	h2	5	h3	10	h2
TALDOR (13476)	FRA	2^*^	h3	7	h1	8	h1	4	h2	12	h1
APACHE (13481)	FRA	null	h2	7	h1	8	h1	3	h2	12	h1
OPATA 85 (13811)	MEX	2^*^	h3	7	h1	9	h1	5	h3	10	h2
SYNTHETIQUE-W7984 (13812)	MEX	null	h6	7	h1	8	h1	N	h4	N	ND
**BLE DE REDON BLANC 1/2 LACHE 1 1 (15658)**	FRA	1	h1	13	h3	16	h1	2	h5	12	h1

*Accessions used for expression studies are shown in bold*.

a *Accession no. in the INRA Triticeae Genetic Resources Collection (http://www6.clermont.inra.fr/umr1095) is given in brackets*.

b *Country names are given as three-letter ISO codes (http://www.unc.edu/~rowlett/units/codes/country.htm)*.

c *Protein coding allele for the x- or y-type HMW-GS identified by SDS-PAGE. HZ, heterozygous*.

d *Haplotype of the promoter for HMW-GS genes. ND, no data*.

### Expression analysis

To quantify HMW-GS gene expression, RNA was extracted from developing grains harvested at 400°C days after anthesis from 13 lines representing the main promoter alleles (Table 1). Lines were cultivated in the greenhouse as described in Ravel et al. (2009). For each of the four lines 964, 1288, 2135, and 4874, four independent biological replicates were obtained. Two independent biological replicates were used for each of the nine remaining accessions. Quantitative-real-time PCR (qRT-PCR) was performed as described in Ravel et al. (2009) using a LightCycler® 480 II sequence detection system and the LightCycler 480 SYBR Green I Master (Roche) according to the manufacturer's instructions. Primer pairs used for qRT-PCR and their amplification efficiency are given in Supplementary Table 2. The specificity of each primer pairs was confirmed by a single peak in the real-time melting temperature curves for each gene.

Amplification plots and predicted threshold cycle values were obtained with LightCycler 480 SW 1.5 software (Roche). Genes coding for glyceraldehyde 3-phosphate dehydrogenase (GAPDH), elongation factor 1 alpha (eF1α), β-tubulin, and 18S RNA were used as internal controls to normalize expression results (Ravel et al., 2009). The geometric mean of control gene expression was calculated so that HMW-GS gene expression could be quantified and normalized also taking into account the efficiency of each primer pair.

### Promoter annotations

Twenty motifs known to participate in the regulation of SSP and two light responsive motifs were selected from the PLACE *cis*-motif database, which contains 469 entries (Table 2; Higo et al., 1999). We included a light responsive (Abox) and a circadian rhythm-responsive (CIACADIANLELHC) motif because diurnal fluctuations in carbohydrate pools and Opaque 2 (O2) binding activity during seed filling may impact SSP synthesis (Ciceri et al., 1997, 1999; Carman and Bishop, 2004). We also added two additional motifs, 5′-AACNNA-3′ and 5′-TATAWA-3′, which were not in the PLACE database. The first motif is able to bind a MYB protein from rice (*Oriza sativa*) belonging to the GAMYB sub-family (Takaiwa et al., 1996). The second motif is the TATA-variant sequence of SSP genes involved in the formation of a transcription initiation complex (Fauteux and Strömvik, 2009; Bernard et al., 2010).

**Table 2:** **Characteristics of *cis*-motifs from PLACE database and bibliographic references used to annotate the promoters of HMW-GS genes**.

**Names**	**Name used in PLACE**	**Sequence**	**Binding transcription factor^a^**	**References**
**DOF**				
DOF core	DOFCOREZM	AAAG	**DOF**	Hammond-Kosack et al., 1993; Yanagisawa and Schmidt, 1999
			SAD, *PBF*, BPBF	
			WPBF	
Pbox1	PROLAMINBOXOSGLUB1	TGCAAAG		Wu et al., 2000; Isabel-La Moneda et al., 2003
Pbox2	300CORE	TGTAAAG		Thomas and Flavell, 1990
Pbox3	−300ELEMENT	TGHAAARK		Marzábal et al., 1998
**bZIP**				
GLM 1	GCN4OSGLUB1	TGAGTCA	**bZIP** *O2*	Albani et al., 1997; Vicente-Carbajosa et al., 1998; Wu et al., 2000
GLM 2	−300MOTIFZMZEIN	RTGAGTCAT	BLZ1, BLZ2	Thomas and Flavell, 1990; Oñate et al., 1999
GLM 3	GLMHVCHORD	RTGASTCAT	SPA	Norre et al., 2002
ACGT core	ACGTATERD1	ACGT		
G-box motif 1	ABREATCONSENSUS	YACGTGGC		Kang et al., 2002; Choi et al., 2000
G-box motif 2	ABRELATERDI	ACGTG		
CAAT	CAATBOX1	CAAT	**bZIP**^b^	Shirsat et al., 1989
**RY-REPEAT**				
RY_core	RYREPEATLEGUMINBOX	CATGCAY	**AB3/VP1** FUSCA3	Fujiwara and Beachy, 1994; Moreno-Risueno et al., 2008; Van Herpen et al., 2008
**AACA MYB**				
AACA motif 1	AACACOREOSGLUB1	AACAAAC	**R2R3-MYB**	Takaiwa et al., 1996; Suzuki et al., 1998; Wu et al., 2000; Diaz et al., 2002
			GaMYB	
			GaMYB	
AACA motif 2	ANAERO1CONSENSUS	AAACAAA		
MYB1AT	MYB1AT	WAACCA		
AACA motif 3	Not referred in PLACE	AACNNA		
**GATA MYB**				
GATA box 1	GATABOX	GATA	**R1MYB**	Rubio-Somoza et al., 2006a
			MCB1	
			MYBS3	
GATA box 2	MYBST1	GGATA	**MYB**	Baranowskij et al., 1994
**OTHERS**				
E box	EBOXBNNAPA	CANNTG	**bHLH**	Chaudhary and Skinner, 1999
CCAAT	CCAATBOX1	CCAAT	**HAP** *CBF*	Albani and Robert, 1995
ESP	ESPASGL01	ACATGTCATCATGT	Not identified	Vickers et al., 2006
TATA-variant	Not refered in PLACE	TATAWA	TATA-box-binding proteins	Fauteux and Strömvik, 2009
CIACADIANLELHC	CIACADIANLELHC	CAANNNNATC		Piechulla et al., 1998
ABox	PALBOXAPC	CCGTCC		

a *Transcription factor families are indicated in bold followed by the name of corresponding transcription factor in maize (italics), barley, wheat (underlined), or other species (italics and underlined)*.

b *Interaction not functionally validated*.

Both strands of the 1-kb region upstream of the start codon for the six HMW-GS genes from cv. Renan retrieved from public databases (DQ537335.1, DQ537336.1, and DQ537337.1 for *Glu-A1*, *Glu-B1*, and *Glu-D1*, respectively; Gu et al., 2006) and the promoter sequences of the five (i.e., all but *Glu-A1-2*) HMW-GS genes obtained in this study for 42 lines (including cv. Renan) of the INRA worldwide hexaploid wheat core collection were annotated using a custom-made PERL program (named PlantPAD) that extracts the name, sequence and coordinates of the motifs and produces a graphical representation of the query sequence on which the starting position of each *cis*-motif is plotted. Based on the assumption that functional *cis*-motifs are conserved among HMW-GS genes, we used PlantPAD to search for co-occurrence of *cis-*motifs in these genes. To build the consensus, the program considers each motif and its coordinates (the position of its first nucleotide relative to the start codon). Any motif that appears at the same coordinates (±5 bp) in all the sequences being annotated is considered to be conserved. As insertion-deletion events (indels) within a sequence cause motifs to shift along the gene, the program also recognizes conserved motifs which appear in all the sequences with the same coordinates plus or minus the shift size (the length of indels). The consensus is then plotted and the distances between conserved motifs corresponding to those found in more than 50% of the sequences are analyzed. Such a consensus is designed to highlight the conserved regulatory regions. This approach was used to analyze separately both sets of orthologous genes and produce a consensus plot for each of them. These consensuses were then used to generate an overall consensus annotation of HMW-GS gene promoters.

### Functional validation

Particle bombardment was performed in developing wheat endosperm to validate *cis-*motifs potentially able to bind bZIP TFs. The promoter of *Glu-B1-1* gene (hereafter termed PrBx7) was amplified and cloned using the primers from cv. Renan given in Supplementary Table 1. A total of 747-bp upstream fragment of the start codon was used. In addition, to assess the role of the distal conserved regulatory regions of this promoter, the 597-bp fragment from the start codon (hereafter termed tPrBx7) was synthesized.

All constructs used for transient expression assay were obtained using Gateway technology (Invitrogen). Three entry clones were used (pDONRP4-P1R, pDONR221, and pDONRP2R-P3). pDONRP4-P1R contained the rice actin promoter, PrBx7 or tPrBx7, while pDONR221 and pDONRP2R-P3 contained a reporter gene (either GUS or GFP, respectively) and the 3′-terminator nopaline synthase gene (3′-NOS). Three expression pDESTR4-R3-based vectors (pAct-GFP, pPrBx7-GUS, and ptPrBx7-GUS) were created. A transient promoter activation assay based on co-bombardment with pPrBx7-GUS or ptPrBx7-GUS and pAct-GFP constructs was performed using immature endosperm from cv. Récital collected at 230°C day after anthesis from plant grown in the greenhouse under optimal growth conditions. Seeds were surface-sterilized and endosperms were carefully isolated. Endosperms were cultured on Murashige and Skoog medium supplemented with maltose (100 g L^−1^) for 2–3 h for subsequent bombardment. Gold particles (0.6 μm in diameter; Bio-Rad) were prepared with 500 ng of a 1:1 molar ratio mixture of pAct-GFP and pPrBx7-GUS or ptPrBx7-GUS. Bombardments were conducted at a distance of 6 cm from the stopping plate using a biolistic helium gun device (PDS-1000, Bio-Rad) with a pressure of 6.21 MPa. Following bombardment, endosperms were incubated for 2 days in the dark at 24°C in a Murashige and Skoog medium supplemented with 3% (w/v) sucrose and 0.15 mM of each of the 20 proteinogenic amino acids. For GUS expression, endosperms were stained with 5-bromo-4-chloro-3-indolyl glucuronide according to Jefferson et al. (1987). Endosperms were observed using a MZ16 F stereomicroscope equipped with a DFC300 FX digital camera (Leica Microsystems) and GUS and GFP activities were determined by counting the number of blue and green cells, respectively. Expression results were normalized by dividing the number GUS foci by the number of GFP foci. For each construct, 10 independent bombardments of eight endosperms each were performed. The pAct-GFP construct was used to determine the efficiency of bombardment as proposed by Eini et al. (2013).

The DNA-binding activity of *cis*-motifs with SPA was studies by EMSA. The SPA protein was expressed in *E. coli* (BL21 AI strain) by cloning *Spa* cDNA into the pDEST17 plasmid vector (Invitrogen) producing pHis-SPA. *Spa* expression was induced with 0.2% (w/v) arabinose for 3 h. Proteins extracts were obtained after re-suspension of the induced cells in a 10 mM Tris buffer (pH 8) containing 6 M urea and 100 mM NaH_2_PO_4_ (10 mL g^−1^ pellet). Purification of the recombinant protein was achieved by loading protein extracts onto a Ni^2+^-NTA resin and bound proteins were eluted in a 10 mM Tris buffer (pH 4.5) containing 6 M urea and 100 mM NaH_2_PO_4_. The eluate was dialyzed against a 10 mM Tris buffer (pH 8.3) containing 2 M urea, 100 mM NaH_2_PO_4_, 100 mM KCl, 0.02% Tween-20, 10% glycerol, and 0.5 mM phenylmethylsulfonyl fluoride (PMSF) for 36 h to renaturate the recombinant protein and then against a 10 mM Tris buffer (pH 7.5) containing 50 mM KCl, 1 mM dithiothreitol, 0.02% Tween™ 20, 10% glycerol, and 0.5 mM PMSF for 16 h. The dialysate was then concentrated with an Amicon 10 kDa filter (Millipore).

DNA oligonucleotides able to bind bZIP TFs (GLM and G-box) used in EMSA are described in Supplementary Table 3. Each single-strand oligonucleotide was labeled using the Biotin 3′ End DNA Labeling Kit (Pierce) following the manufacturer's instructions and hybridized for 30 min at the annealing temperature of the probes. The labeled dsDNA probe (20 fmol) was incubated with 560 ng to 4 μg of recombinant His-SPA protein in 20 μL of a binding buffer containing 10 mM Tris (pH 7.5), 2 mM dithiothreitol, 100 mM KCl, 10% glycerol, 0.05% nonyl phenoxypolyethoxylethanol, 2 mM ethylenediaminetetraacetic acid, 100 ng μL^−1^ poly(dI.dC), 250 ng μL^−1^ fish sperm DNA, 0.5 mM PMSF for 30 min at room temperature. DNA-protein complexes were analyzed by non-denaturing 6% polyacrylamide gel electrophoresis in a 45 mM Tris, 45 mM Borate, and 1 mM ethylenediaminetetraacetic acid buffer (pH 8.3). After separation (100 V, 1 h at 4°C), gels were electroblotted to nylon membranes using the same buffer (380 mA, 45 min at 4°C). The biotin end-labeled DNA was detected using streptavidin, horseradish peroxidase conjugate following the manufacturer's instructions (LightShift Chemiluminescent EMSA kit, Pierce).

### Statistical analyses

All statistical analyses were done using R 3.0 software (R Core Team, 2013). The normality of and homogeneity of variances of expression data were tested by the Shapiro–Wilk and Bartlett's tests, respectively. Depending on the results of previous analyses, expression data were submitted to non-parametric or parametric variance analysis with the Kruskal–Wallis or the general linear model procedure. Multiple comparison tests between groups after Kruskal–Wallis tests were done with the Kruskalmc function while the Student–Newman–Keuls test was used to compare means after the general linear model procedure. The Kruskal–Wallis and Student–Newman–Keuls tests used were those available in the R “agricolae” (version 1.1-8) package (De Mendiburu, 2014), all other tests were done using the R “Stats” (version 2.15.3) package. All the data were used in a first analysis based on a model with one factor (gene). In a second step, analyses were carried out gene per gene to study the promoter haplotype factor.

To analyze the differences in expression of HMW-GS genes and haplotypes one-way ANOVA were performed. First, an ANOVA with the gene as the main factor was carried out. The four lines with the null allele at *Glu-A1-1* and the line with protein allele 7 overexpressed (7OE) at *Glu-B1-1* were excluded from this analysis to avoid bias. Secondly, ANOVAs with the promoter haplotype as the main factor were performed for each gene (including the null allele at *Glu-A1-1*).

Differences in normalized expression from transient expression assays were analyzed using *t*-test. All statistically significant differences were judge at 5%.

## Results

### The variability of the promoter is not systematically connected with phenotypic variability

The variability in the nucleotide sequence of the promoters of the five HMW-GS genes was extensively studied by sequencing a set of 42 lines representative of the diversity present in the INRA worldwide hexaploid wheat core collection. The following results deal with the noncoding DNA region upstream of the start codon given that for HMW-GS genes the transcription start site (TSS) is about 60 bases upstream of the start codon for translation. In some cases, the hybridization sites of reverse primers were downstream of the start codons, so the sizes of the upstream fragments studied ranged from 467 to 1138 bp. A total of 36 single-base changes, 2 single-base insertion-deletions (indels) and 1 larger indel were identified in an average of 3858 bp promoter sequence per line (Table 3, Supplementary Table 4). These specific regions have an average of one polymorphism every 100 bases. The number of polymorphisms varied between promoters. *Glu-B1-2* promoter has one polymorphism every 58 bp, threefold more frequently than the *Glu-D1-2* promoter, which has one polymorphism every 145 bp. One large deletion of 54 bp spanning from 291 to 344 upstream of the start codon in the *Glu-B1-1* promoter was observed in two lines (accession nos. 4901 and 15658). Thus, nucleotide diversity estimated by the mean pairwise difference (π) varied from one promoter to another, ranging from 1.5 × 10^−3^ for *Glu-D1-1* to 3.0 × 10^−3^ for *Glu-B1-1*. Except for *Glu-D1-2*, we observed that the nucleotide diversity (π) and the number of segregating sites (θ) are about equal in values as confirmed by the non-significant Tajima's D statistic (Table 3). This suggests that there has been no particular pattern of selection in these regions.

**Table 3:** **Number of electrophoretic alleles revealed by SDS-PAGE, haplotype and diversity statistics for the promoters of five HMW-GS genes from 42 accessions of the INRA worldwide hexaploid wheat core collection**.

**Genes**	**No. of electrophoretic alleles^a^**	**Promoter length (bp)**	**No. of polymorphic sites/No. of indels^b^**	**No. of haplotypes^c^**	**Haplotype diversity, Hd**	**Nucleotide diversity, π**	**No. of segregating sites, θ**	**Tajima's statistic**
*Glu-A1-1*	3	878	7 (4)/0	6 (3)	0.71	1.51 × 10^−3^	1.60 × 10^−3^	NS^d^
*Glu-B1-1*	7	747	10 (3)/1 (0) *54*	5 (2)	0.34	2.67 × 10^−3^	3.16 × 10^−3^	NS
*Glu-B1-2*	6	465	7 (2)/1 (0) *1*	5 (1)	0.45	3.03 × 10^−3^	3.03 × 10^−3^	NS
*Glu-D1-1*	4	667	5 (3)/0	5 (2)	0.49	1.48 × 10^−3^	1.52 × 10^−3^	NS
*Glu-D1-2*	2	1163	7 (0)/1 (0) *1*	2 (0)	0.43	2.93 × 10^−3^	1.39 × 10^−3^	*P* < 0.05

a *Protein coding allele for the x- or y-type HMW-GS identified by SDS-PAGE*.

b *The number of singletons (i.e., a polymorphism found in a single line) is given in brackets; the size of indels is indicated in italics*.

c *The number of haplotypes including a single line is indicated in brackets*.

d *NS, not significant*.

**Figure 1:**
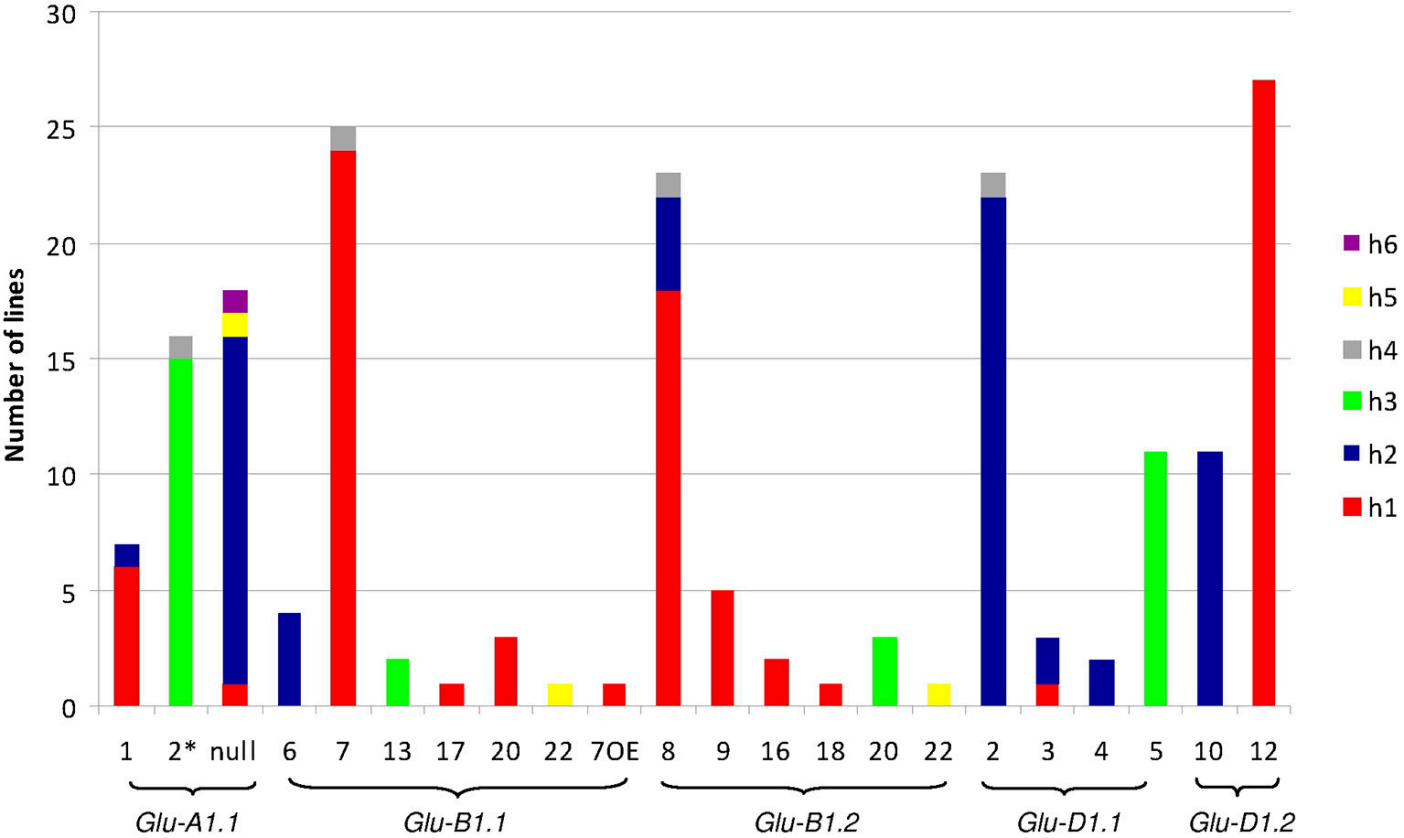
**Number of lines of each haplotype of HMW-GS gene promoter for all electrophoretic forms of HMW-GS present in the set of 42 lines studied**. The promoter haplotypes are named h1 to h6. The same color is used for the same haplotype number of a given gene promoter. Although the color is identical for all h1 haplotypes, their sequences differ (e.g., the sequences of haplotype h1 at *Glu-A1-1* and *-B1-1* are different).

The polymorphisms are linked by a high level of linkage disequilibrium (data not shown). Therefore, for all loci, most of the lines clustered into two main haplotypes with the remaining haplotypes being generally represented by single lines. Notably, the number of haplotypes found for each promoter fits the number of protein coding alleles for *Glu-D1-2* only (Table 1, Figure 1). For *Glu-B1-1*, we observed more protein coding alleles than promoter haplotypes. For the three other *Glu1* genes, we observed more promoter haplotypes than protein coding alleles. Each electrophoretic allele, except for *Glu-B1-2* alleles, tends to have a more-frequent promoter haplotype (Figure 1).

### The variability of the HMW-GS gene promoter is often connected with the level of gene transcription

To assess whether the gene transcrition level is influenced by the promoter haplotype of each HMW-GS gene, HMW-GS transcripts were quantified at 400°C days after anthesis for 13 lines by qRT-PCR (Table 1). The five HMW-GS genes had different levels of transcription (*P* = 2 × 10^−16^). On average, *Glu-B1-1* and *Glu-D1-1* showed a higher level of transcription than the remaining genes, while *Glu-D1-2* was expressed at lower levels (Table 4). The two x-type HMW-GS genes were expressed up to 10-fold higher than the genes coding the y-type. The transcription of *Glu-A1-1* was intermediate.

**Table 4:** **Comparison of the transcription levels of HMW-GS genes at 400°C days after anthesis for 13 lines of INRA worldwide hexaploid wheat core collection**.

**Genes**	**No. of lines^a^**	**RNA expression levels^b^**
*Glu-A1-1*	9 (22)	56.70 ± 3.99 (B)
*Glu-B1-1*	12 (32)	83.64 ± 7.65 (A)
*Glu-B1-2*	13 (34)	17.14 ± 0.97 (C)
*Glu-D1-1*	13 (34)	83.69 ± 5.02 (A)
*Glu-D1-2*	13 (34)	8.063 ± 0.42 (D)

*Data are means ± 1 SE*.

a *The number of data points is indicated in brackets*.

b *Different letters in brackets indicate a significant difference (α = 5%) calculated according to a Kruskal–Wallis non-parametric test followed by the Kruskal multiple comparisons test*.

Among the four accessions with the null allele at *Glu-A1-1*, three harbor the h2 promoter haplotype and one the h5 haplotype. These two haplotypes differ by only one single nucleotide polymorphism (SNP) and their transcription was close to zero (Table 5). The transcription of the two other promoter haplotypes for *Glu-A1-1* were not different (*P* = 0.95). One line (accession no. 8058) harbors the h2 haplotype but has the protein allele 1 and had a transcription close to that of the h1 and h3 promoter haplotypes. For *Glu-B1-1*, once the line with the Bx7OE protein allele was discarded, the promoter haplotype effect was significant (*P* = 0.014). The transcription for the h1, h3, and h4 haplotypes was similar and, on average, 2.6-fold higher than that for haplotype h2 (Table 5), which only includes the Bx6 protein allele (Figure 1). The line with the Bx7OE protein allele has the h1 promoter haplotype, as most of the BX7 protein alleles, but it expressed *Glu-B1-1* at a level (195.33 ± 29.25, *n* = 2) twice that of Bx7 lines. For *Glu-B1-2*, the haplotype effect was significant (*P* = 0.023) and transcription from h1 was higher than from h3 (Table 5). For this gene, the promoter haplotypes were not linked with separate protein alleles (Figure 1). The RNA expression of the *Glu-D1-1* and *Glu-D1-2* alleles was not influenced by their promoter haplotypes (data not shown).

**Table 5:** **Multiple comparison of the mean levels of RNA expression from promoter alleles of HMW-GS genes at 400°C days after anthesis**.

**Genes**	**Promoter haplotype**	**No. of lines^a^**	**RNA expression levels^b^**
*Glu-A1-1*	h1	1 (2)	58.25 ± 1.83 (B)
	h2 (null)^d^	3 (10)	1.40 ± 0.18 (A)
	h2 (1)^d^	1 (2)	57.38 ± 7.70 (B)
	h3	6 (16)	58.56 ± 5.19 (B)
	h5	1 (2)	1.41 ± 0.32 (A)
*Glu-B1-1*^c^	h1	7 (20)	102.27 ± 9.08 (A)
	h2	3 (8)	35.36 ± 3.43 (B)
	h3	1 (2)	87.23 ± 7.35 (A)
	h4	1 (2)	86.94 ± 11.50 (A)
*Glu-B1-2*	h1	8 (22)	18.96 ± 1.24 (A)
	h2	3 (8)	14.74 ± 1.28 (AB)
	h3	2 (4)	11.89 ± 1.45 (B)

*Data are means ± 1 SE*.

a *The number of data points is indicated in brackets*.

b *Different letters in brackets indicate a significant difference (α = *5*%) calculated according to a Kruskal–Wallis non-parametric test followed by the Kruskal multiple comparisons test*.

c *The line accession no. 3358 with the 7OE allele was discarded*.

d *For Glu-A1-1 haplotype 2, results for the null and 1 protein alleles (indicated in brackets) were treated as two different haplotypes in the ANOVA*.

These results highlight different RNA expression levels for different HMW-GS genes and, for three HMW-GS genes, the effects of the promoter haplotype. Thus, differences in the regulation of these genes might stem from the organization of the *cis*-motifs in their promoters.

### Common *cis*-motifs organization of HMW-GS gene promoters

To analyze the organization of *cis*-motifs in HMW-GS gene promoter, we first searched for similar patterns in the 1-kb promoter region of the six HMW-GS genes of cv. Renan, as HMW-GS genes have similar expression patterns during development and in response to environmental factors. We then compared the consensus organization of *cis*-motifs found for cv. Renan with that found for the haplotypes of each gene to relate differences in *cis*-motifs organization with differences in gene expression.

In all six HMW-GS gene promoters of cv. Renan we found all the 24 *cis*-motifs we annotated but the Pbox2 and ESP motifs. Most of these motifs were annotated several times and a total of 44 (for *Glu-B1-2*) to 54 (for *Glu-D1-2*) *cis*-motifs per gene were annotated. All the *cis*-motifs able to bind all TFs known to regulate the expression of SSP genes were present, but the typical bipartite endosperm box was not found. The number of *cis*-motifs found was over-estimated as the sequences of a few motifs (Table 2) were nested within some others. Most of the nested *cis*-motifs bind TFs of the same family (Table 2). Therefore, we took into account only the longest motif where nested motifs were predicted, which reduced the number of *cis*-motifs per gene by 15–24%. Motifs able to bind MYB TFs (GAMYB, MCB1, MYBS3) were predominant, with 9–14 *cis*-motifs per gene, followed by motifs able to bind bZIP TFs, with 9–13 *cis*-motifs per gene, and DOF TFs (PBF, SAD), with 4–8 *cis*-motifs per gene. The CAAT *cis*-motif accounted for about two-thirds of the total number of *cis*-motifs able to bind bZIP TFs (Table 6).

**Table 6:** **Number of motifs in the upstream 1000-bp region of the six HMW-GS genes from the hexaploid wheat cv. Renan**.

**Motif sequence**	**Binding transcription factor**	***Glu-A1-1***	***Glu-B1-1***	***Glu-D1-1***	***Glu-A1-2***	***Glu-B1-2***	***Glu-D1-2***
**DOF**							
AAAG	DOF	6	4	4	7	4	5
TGCAAAG	DOF		1	1	1		1
TGHAAARK	DOF	1		2			1
**bZIP**							
ACGT^a^	bZIP		1	1	2	3	2
ACGTG^a^	bZIP	1					
RTGAGTCAT^b^	bZIP				1	1	
TGAGTCA^b^	bZIP	2	2	2			2
YACGTGGC^b^	bZIP		1	1	1	1	1
CAAT	bZIP^c^	7	9	7	6	6	7
**AACA MYB**							
AAACAAA	R2R3-MYB	2	1	2	1	1	1
WAACCA	GAMYB			1	2	2	1
AACNNA	R2R3-MYB	1	1	1	1	1	1
**GATA MYB**							
GGATA	MYB/R1MYB	8	7	9	7	8	7
**RY REPEAT**							
CATGCA	AB3/VP1	2	2	2	3	1	2
**OTHERS**							
CANNTG	bHLH	4	4	4	7	5	4
CCAAT	HAP	2	3	2	2	2	2
TATAWA	TATA-box-Binding Proteins	1	1	1	1	1	1
CAANNNNATC		1	2	2	2	2	2
CCGTCC		1	1		1		1
Total		39	40	42	45	38	41

a *Related to GLM*.

b *Related to G-box motifs*.

c *Interaction not functionally validated*.

**Figure 2:**
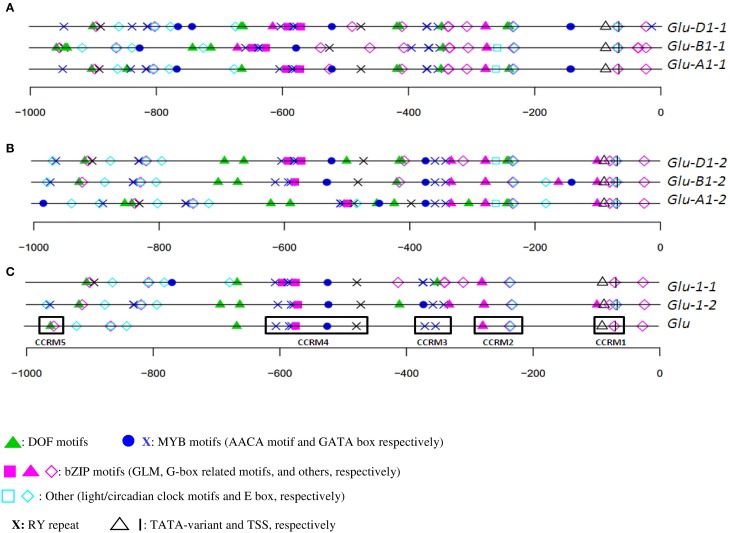
***In silico* annotation of HMW-GS promoters of cv. Renan**. Positions are indicated relatively to the start site. Sequences were obtained from public databases. **(A)** x-type *Glu-A1-1*, -*B1-1*, and -*D1-1* homoeologs; **(B)** y-types *Glu-A1-2*, -*B1-2*, and -*D1-2* homoeologs; and **(C)** consensus annotations of all orthologous set of sequences (*Glu-1-1* and *Glu-1-2* for the x- and y-type HMW-GS promoters, respectively), and of paralogous sequences (*Glu*) with its five conserved *cis*-regulatory modules CCRM1 to CCRM5.

The organization of orthologous promoters from cv. Renan showed few differences (Figures 2A,B) on the plus strand. For x-type HMW-GS genes, the organization was well conserved between 0 and −400 (nucleotide position relative to the start site). The TATA-box was at −90. A few differences were detected like an AACA motif at −144 in *Glu-A1-1* and *-D1-1*, which was absent in the orthologous B sequence. Between −400 and −1000, the organization was also well conserved but a 55-bp insertion in the *Glu-B1-1* promoter shifted the *cis*-motif upstream (i.e., at a more negative nucleotide position) of the insertion. Interestingly, we discovered a composite box named the GLM-GATA box. This box includes two repeated units, each of them made of a GATA motif and a GLM separated by a third GGATA motif. The relative positions of the constitutive *cis*-motifs in this box were conserved among the three orthologous sequences of cv. Renan (Figure 2). An ACGT motif was present a few bases upstream of this box in the B and D sequences. About 50 and 200 nucleotides upstream of this box, a DOF core motif (AAG) and an AACA motif (able to bind R2R3-MYB TFs), respectively, were detected in all the homoeologs. Downstream of this box, we found an AACA motif able to bind R2R3-MYB and the RY repeat.

Similar observations were made for the y-type sequences (Figures 2B,C). *Cis*-motifs organization presented many similarities between positions 0 and −400, although the promoter of *Glu-B1-2* includes some additional motifs at about position −150. In addition, the entire composite GLM-GATA box was lacking in the promoters of *Glu-A1-2* and -*B1-2*, the latter containing only a single copy of the GLM. None of these three sequences included the ACGT motif near the GLM-GATA found in the x-type HMW-GS gene promoters. We observed a composite motif at position −400, which was conserved in these three homoeologous sequences, composed of a G-box and three consecutive MYB motifs (two GATA and one AACA motifs). At about position −400, a deletion shortened the distances between the motifs at −400 and the adjacent ones on the *Glu-A1-2* promoter causing a deletion of a few motifs. For the three y-type homoeologous genes, an RY repeat and an AACA motif (binding R2R3-MYB) were located between position −400 and the GLM-GATA box.

The overall consensus generated from all HMW-GS genes of cv. Renan (Figure 2C) consisted of 21 motifs including motifs able to bind all the TFs known to regulate SSP synthesis so far. They were organized into five CCRMs. CCRMs were numbered from 1 to 5 from the start codon and composed of two to five *cis*-elements. As expected, CCRM1, a few nucleotides upstream of the TSS, was composed of the TATA-box variant and the CAAT motif. CCRM2 included a G-box-like motif and a CAAT motif, nested into an E-box (CANNTG), while CCRM3 clustered two GATA boxes. CCRM4 was the most interesting module. It included the incomplete GLM-GATA box, an AACA motif and the RY repeat. The GLM-GATA box was incomplete because of a missing GLM in the cv. Renan allele at *Glu-B1-2*. The fifth module, CCRM5, has a DOF motif and a CAAT box nested into an E-box and is located between positions −900 and −1000 in all promoters. A few bases downstream of CCRM5, E-boxes and circadian motifs were conserved. No typical bipartite endosperm box was detected. On the minus strand, we noted an over-representation of the DOF core AAAG motif (data not shown).

**Figure 3:**
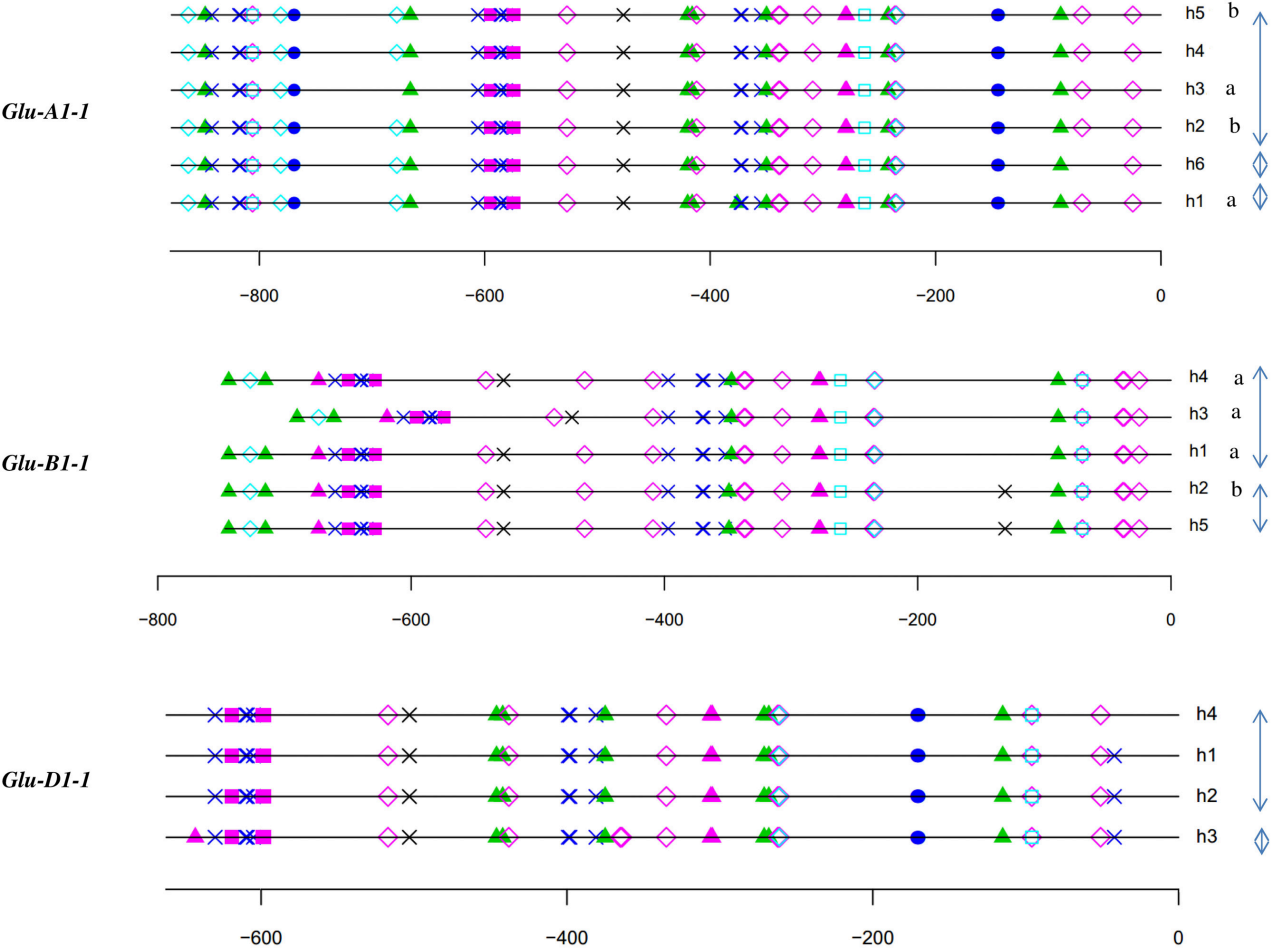
***In silico* annotation of the x-type HMW-GS gene promoter haplotypes**. Positions are indicated relative to the transcription start site. Sequences were obtained from a set of 42 lines representative of the genetic diversity of the INRA worldwide hexaploid wheat core collection. For each gene, the haplotype of the promoter is indicated by the letter h followed by the number of the haplotype. Letters a and b indicate the significantly different groups for the mean of expression for haplotypes studied by qRT-PCR. Clusters of haplotypes differing by one polymorphism are shown with gray arrows on the right. See the key to Figure 2 for descriptions of *cis*-motif symbols.

**Figure 4:**
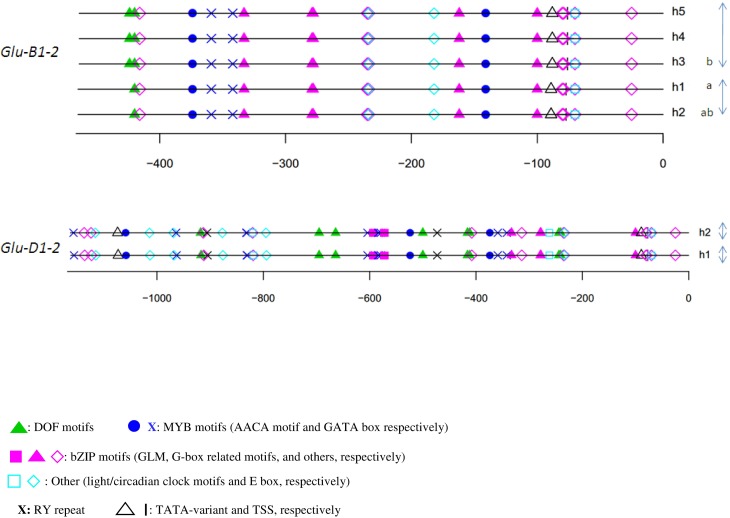
***In silico* annotation of the y-type HMW-GS gene promoter haplotypes**. Positions are indicated relative to the transcription start site. Sequences were obtained from a set of 42 lines representative of the genetic diversity of the INRA worldwide hexaploid wheat core collection. For each gene, the haplotype of the promoter is indicated by the letter h followed by the number of the haplotype. Letters a and b indicate the significantly different groups for the mean of expression for haplotypes studied by qRT-PCR. Clusters of haplotypes differing by one polymorphism are shown with gray arrows on the right.

For each HMW-GS gene, except *Glu-B1-1*, the annotation of haplotypes was almost identical (Figures 3, 4). Three groups were observed for *Glu-B1-1*. Haplotypes h2 and h5 have identical annotations, but compared to the other haplotypes, they contain an additional RY repeat at position −160. The second group contains h1 and h4, which are distinct from h3 because of an indel. Distances between motifs upstream and downstream of position −400 are therefore shorter in h3 than in the other haplotypes. In addition, a bZIP motif present in the insertion is deleted in h3. The haplotype h3 of *Glu-D1-1* promoter differs from other haplotypes as it has two additional bZIP motifs, one being a G-box.

The relative position of the GLM-box was conserved in all haplotypes of the three orthologous sequences of the x-type HMW-GS genes (Figure 3) and the y-type *Glu-D1-2* gene (Figure 4). For *Glu-B1-2*, the region sequenced in this study did not cover the GLM-GATA box (Figure 4), but the analysis of *Glu-B1-2* promoter sequences of cv. Chinese Spring (KC20630) and Xiaoyan 54 (EU137874), available in public databases, shows that, in these cases, the relative position of the GLM-box is also conserved in this gene (data not shown).

## The GLM-GATA box is involved in the regulation of *GLU-B1-1* expression

**Figure 5:**
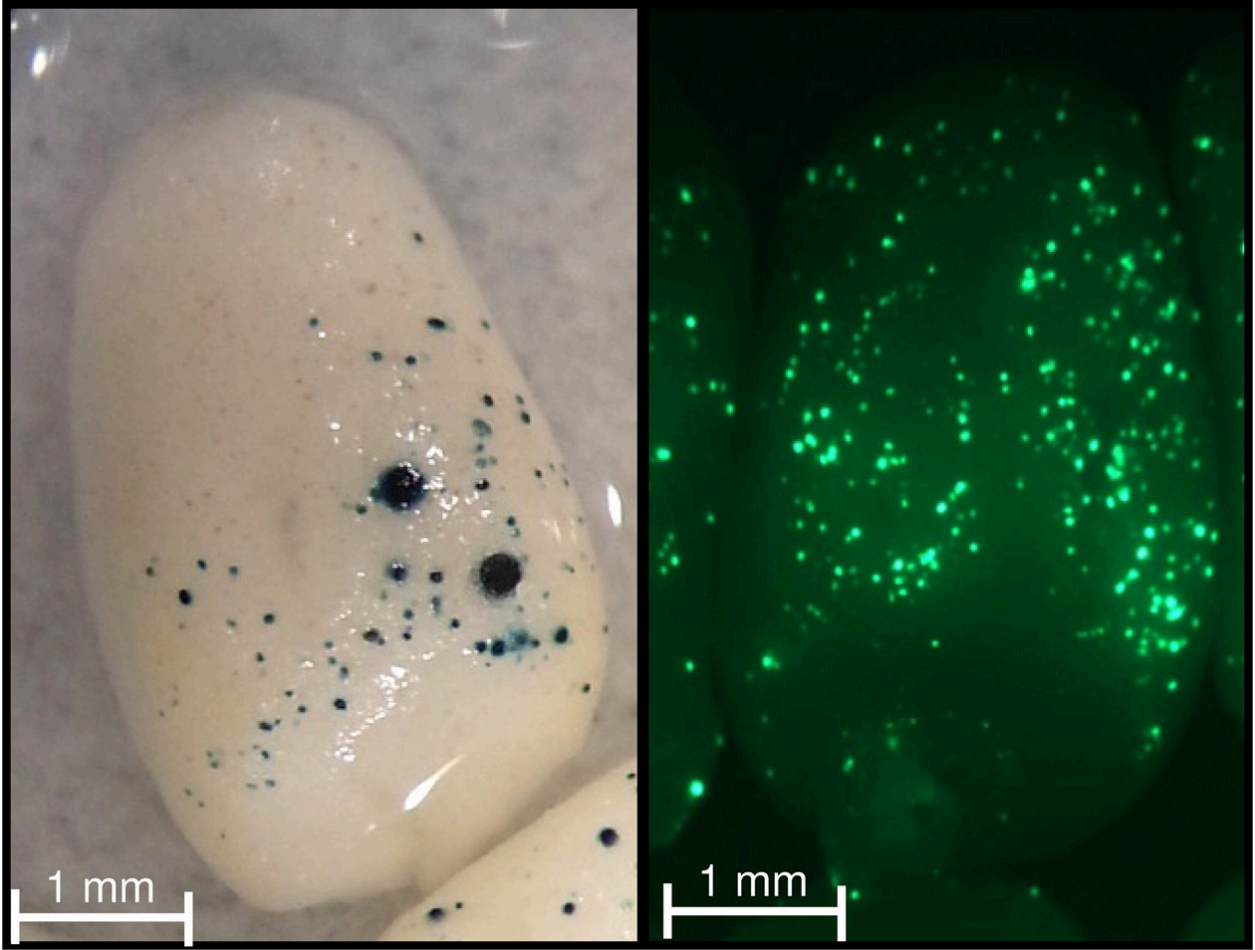
**GUS and GFP activities in wheat immature endosperm**. Immature endosperm was co-bombarded with the pPrBx7-GUS and pAct-GFP constructs. Note the blue (bottom panel) and green (top panel) foci across the dorsal surface.

**Figure 6:**
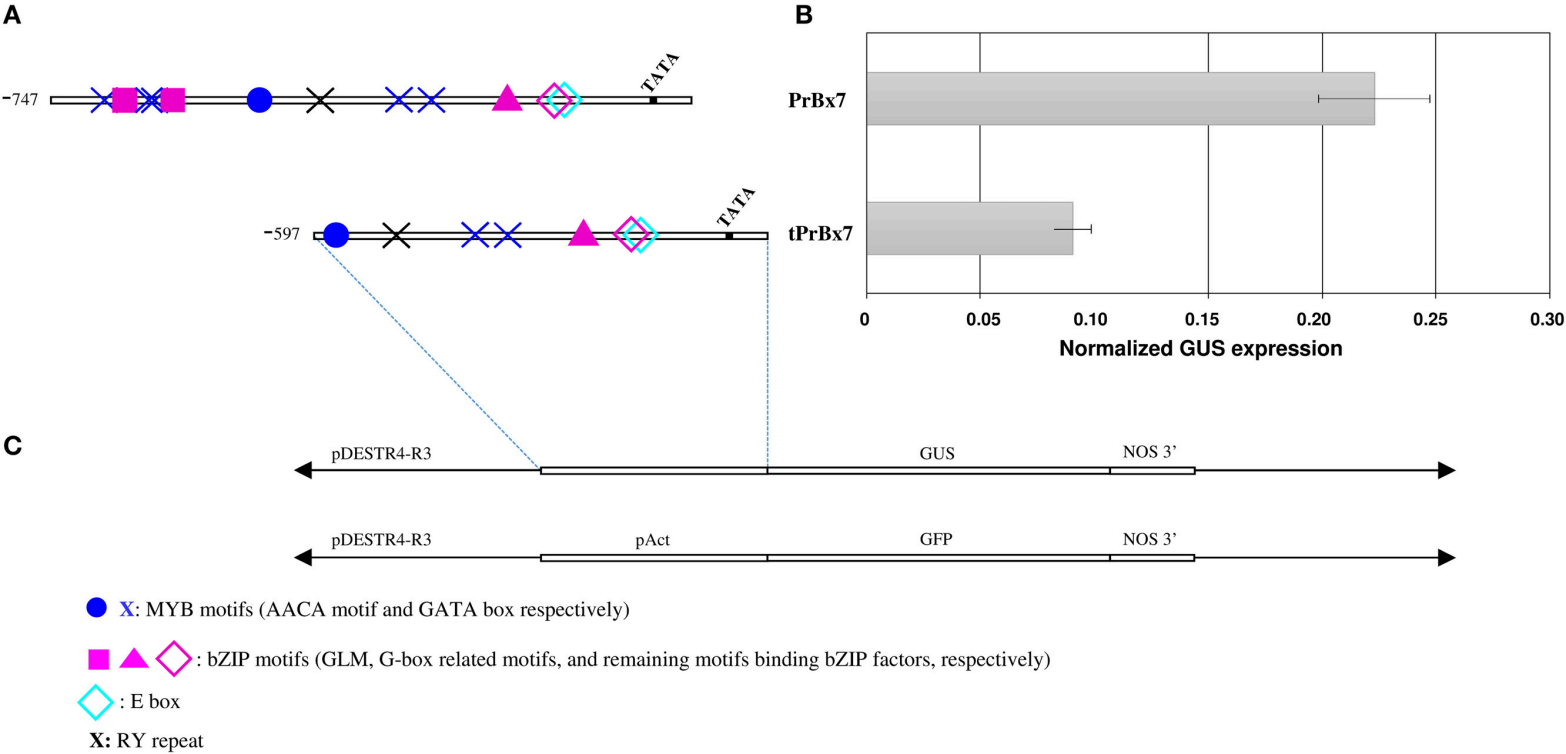
**Activity of *Glu-B1-1* gene promoter from cv. Renan (Bx7) in immature wheat endosperms using a transient expression assay. (A)** Schematic representation of the constructs used. The TATA box and nucleotide positions relative to the start codon and corresponding to deleted region are indicated. Putative *cis*-regulatory elements, E-box (−259), G-box (−277), GATA box (−658, −638, −633, −368, and −350), RY motif (−525), AACA motif (−233), GLM1 and GLM2 (−647 and 626, respectively) are shown. **(B)** Normalized GUS expression of the corresponding promoters in transiently transformed endosperms. Data are the mean ± 1 SE. for *n* = 10 independent bombardments. **(C)** Schematic representation of the GUS constructs used for transformation.

To investigate the involvement of the GLM-GATA box in the regulation of HMW-GS gene expression, we analyzed the effect of the 5′ deletion from positions −747 to −597 (fragment carrying the GLM-GATA box) by transient expression experiment (Figures 5, 6). The deletion of the GLM-GATA box reduced normalized GUS expression by 59%.

**Figure 7:**
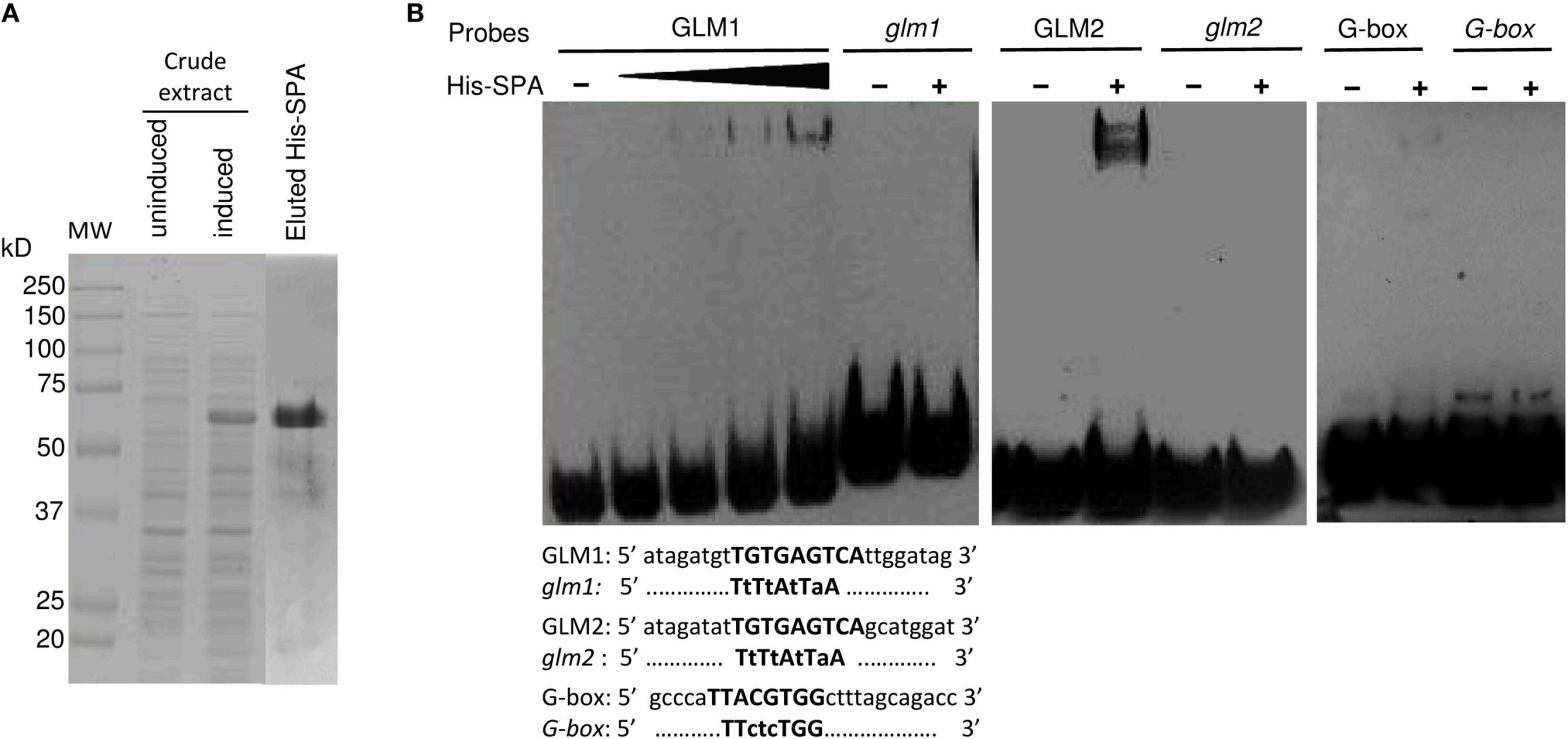
**Binding of recombinant SPA protein with the probes derived from the *Glu-B1-1* gene promoter. (A)** Expression and purification of recombinant His-SPA protein. Crude extracts from uninduced and induced bacteria harboring the pHis-SPA expression vector and the eluted protein were resolved on an SDS-polyacrylamide gel. The molecular mass markers are indicated at left in kilodaltons. **(B)** EMSA of the recombinant SPA protein with the 25-bp biotin-labeled, GLM1 (−647), GLM2 (−626), and G-box (−227) probes derived from the *Glu-B1-1* gene promoter and their mutated versions *glm1*, *glm2*, and *G-box*. The sequences of the oligonucleotides used as probes are shown with the GLM1, GLM2, and G-box in bold; identical residues are represented by dots, and mutated bases are shown in lowercase.

To verify the potential binding activity of the two GLMs (GLM1 and GLM2 at positions −647 and −626, respectively) present in the GLM-GATA box of the *Glu-B1-1* gene promoter, we performed EMSAs with synthetic oligonucleotides and a recombinant SPA protein expressed as a His fusion in *E. coli* (Figure 7). We also determined the *in vitro* binding of SPA to the G-box motif, which was previously shown to bind bZIP proteins (Norre et al., 2002). As shown in Figure 7A, arabinose treatment induced expression of a protein of 50–75 kDa that was not present in uninduced cell extracts. The apparent size of the recombinant protein determined by SDS-PAGE was larger than the expected 48 kDa molecular mass of the His-tagged SPA fusion protein. A similar apparent increase in size on SDS gels was already reported by Albani et al. (1997) in their study of SPA. The recombinant His-SPA protein was purified to near homogeneity and used for binding assays. A DNA-protein complex was clearly observed with the GLM2 motif, while the shifted band detected for the GLM1 and the G-box was considerably fainter (Figure 7B). No shifted band was observed when incubation was carried out with the mutated probes (*glm1*, *glm2*, and *G-box*). DNA-binding affinity of the recombinant protein seems to be greater with the GLM2 probe than the other probes tested.

## Discussion

Here we characterized and annotated wheat HMW-GS gene promoters. The expression of these genes in developing grain was quantified by qRT-PCR and the correlations between the variability in expression and the variability in predicted *cis*-element motifs of the corresponding promoter were also analyzed. We considered regions of 467–1138 bp upstream of the start codon. In *Arabidopsis thaliana*, based on the density of polymorphisms in gene upstream regions, functional promoters require 250–500 nucleotides upstream of the TSS (Korkuć et al., 2014). Under the assumption that promoter length is conserved, the lengths of the regions surveyed here provide a reasonable coverage of functional SSP gene promoters in wheat. Moreover, we analyzed the role of the GLM-GATA box of the *Glu-B1-1* gene promoter by transient expression assay and evaluated the functionality of the *cis*-motifs reported to bind bZIP TFs.

### Variability of HMW-GS promoter haplotypes cannot be used directly to screen for electrophoretic alleles

In *A. thaliana*, the nucleotide variability in promoters varies depending on the function of their downstream gene (Korkuć et al., 2014). It is higher for genes involved in adaptive processes and transcriptional regulation than for genes involved in housekeeping functions. In wheat, the diversity of promoters is not widely documented so far. The range of nucleotide diversity observed for HMW-GS promoters, approximately one polymorphism every 100 bases, is comparable to that reported for the SPA promoter (Ravel et al., 2009), but is higher than the overall level of polymorphism of one SNP every 212 nucleotides reported for promoters of other genes (Ravel et al., 2006). Although upstream gene regions are somewhat constrained as they are involved in gene regulation, they are reported to show higher variability than coding regions. Constraints most likely apply to *cis*-regulatory elements (Korkuć et al., 2014). As they affect short regions, mutations could occur with little or no incidence, whereas the entire coding sequence has to withstand greater constraints. In addition, the modular organization of *cis*-elements, together with their redundancy, may buffer the effects of mutations (reviewed by Purugganan, 2000). These reasons probably explain why the diversity is higher in promoter regions than in coding sequences. As usually reported (e.g., Chao et al., 2009), the level of diversity was the lowest in HMW-GS sequences from the D genome with 1 polymorphism every 145 base for *Glu-D1-2*, whereas the highest level of diversity was observed for HMW-GS promoters from the B genome with, on average, one polymorphism every 60 bases.

SDS-PAGE is still routinely used for characterization of HMW-GS alleles. Developing diagnostic SNPs to identify electrophoretic forms of HMW-GS from any part of young plants would be a valuable tool to support breeding for improved flour quality. However, there are up to four haplotypes promoter sequences per electrophoretic allele or only one haplotype for several alleles. Anderson et al. (1998) already reported two different alleles for the Bx7 promoter. The promoter haplotypes perfectly match the protein alleles only for *Glu-D1-2*. Currently, the identification of a set of SNPs from the other HMW-GS promoter sequences as a shortcut to distinguish between different protein forms is not possible, so the search for diagnostic SNPs needs to continue.

### A minimal framework for the transcriptional regulation of HMW-GS genes is revealed

We screened for *cis*-elements known to regulate SSP synthesis among all the HMW-GS gene promoters of cv. Renan. By annotating these promoters we found that they had a few regulatory elements in common, mostly organized into five CCRMs. Since HMW-GS genes show similar patterns of spatial and temporal expression, these common *cis*-elements might be involved in their global regulation and consequently may provide a minimal regulatory framework needed for the developmental and environmental (i.e., in response to nitrogen supply) regulation of HMW-GS gene expression. Like the long endosperm box described in some LMW-GS gene promoters, which consists of two repeats of the endosperm box (Albani et al., 1997; Juhász et al., 2011), the GLM-GATA box described here for the first time is also formed by two motifs (GATA and GLM) repeated twice in most of the promoters of HMW-GS. Our results demonstrate that the GATA-GLM box has an activator effect. Its two GLMs were able to bind SPA and were thus functional *cis*-motifs. GATA and GLM motifs are reported to bind R1MYB and bZIP TFs. Modules able to bind MYB and bZIP proteins belong to the seven best-known combinations of *cis*-motifs and are also very well represented in *A. thaliana* and poplar promoters (Ding et al., 2012). However, these modules generally bind R2R3-MYB TFs and thus include AACA rather than GATA motifs.

This GLM-GATA box is included in a CCRM with an AACA motif and a RY repeat. Notably, this conserved module is able to bind all the *cis*-motifs reported to regulate SSP synthesis. The minimal regulatory framework contains no P-box like those responsible for endosperm-specific expression of LMW-GS genes. However, several motifs have been reported to be involved in endosperm-specific expression like the CAAT, AACA and ESP motifs (Shirsat et al., 1989; Takaiwa et al., 1996; Vickers et al., 2006). The minimal regulatory framework also contains CAAT motifs. Possibly the G-box acts like the GLM in rice, which has been demonstrated to be an essential element conferring endosperm-specific expression, while P-box and AACA motifs are involved in quantitative regulation (Wu et al., 2000). In addition, the HMW-GS framework contains motifs involved in circadian rhythms. The E-box, which is able to bind bHLH and other TFs, has been reported to be involved in circadian transcriptional rhythms (Seitz et al., 2010), although exactly the same E-box sequence (5′-CATCTG-3′) was not found in the HMW-GS promoters.

Previous reports demonstrated that the 277 bp immediately upstream of the TSS are sufficient for temporal and tissue-specific regulation (Halford et al., 1989; Norre et al., 2002). There is also strong evidence indicating that mutations in this region are responsible for the silencing of *Glu-A1-2* (Halford et al., 1989). However, we did not find any mutation that could alter *cis*-motifs known to be involved in SSP gene regulation. In addition, the mutations specific to *Glu-A1-2* promoter did not create or alter any of the *cis*-motifs of the PLACE database. This suggests that this region may contain *cis-*motifs not yet known or that the mutations encountered in *Glu-A1-2* promoter may alter the affinity of *cis*-motifs identified for their respective TF. More precisely, this fragment contains CCRM1 and CCRM2. The latter includes the G-box found in the *Glu-D1-1* promoter and described by Norre et al. (2002) as being necessary and sufficient for expression. This box has been demonstrated to bind bZIP factors (Norre et al., 2002). CCRM2 also includes the 5′ part of the enhancer element found by Thomas and Flavell (1990), which confirms its important role. Thus, both functional validation and *in silico* analysis confirm the key role of this G-box in regulating the expression of HMW-GS genes. However, the level of expression of HMW-GS genes can be increased by adding more extensive flanking DNA (Anderson et al., 1998; Lamacchia et al., 2001), suggesting the presence of additional more distal *cis*-regulatory elements to the ones we found. This is in agreement with our results, which show a higher level of activity when the promoter of *Glu-B1-1* contained the distal GATA-GLM box. In addition, the DNA-binding affinity of SPA with one of the two GLMs of the GATA-GLM box was higher than that observed with the G-Box, suggesting a stronger role of this motif.

### Differences in expression are only partially explained by annotated *cis*-elements

Our annotation strategy revealed differences at several levels: between paralogous HMW-GS genes, between orthologous HMW-GS genes and between haplotypes of a given HMW-GS gene. To investigate whether different annotated motifs induce quantitative differences in expression, we measured the level of expression from several HMW-GS promoter haplotypes. The expression of x-type gene transcripts was significantly greater than that of y-type transcripts with *Glu-B1-1* and *-D1-1* transcripts being the most expressed, *Glu-A1-1* intermediate and the two remaining genes the least abundant. This result is partially supported by GeneChip® hybridization experiments, which showed that *Glu-B1-1* is the most highly expressed HMW-GS gene in cv. Hereward (Shewry et al., 2009). However, comparing these two sources of results is not straightforward as HMW-GS probe sets cross-hybridize making it difficult to quantify the level of gene expression precisely, and only one wheat line was tested. Comparison of the consensus *cis*-motif framework of *Glu-1-1* with that of *Glu-1-2* showed several differences, which would be expected to impact their expression. Particularly, all *Glu-1-1* promoters contain an additional motif able to bind GAMYB upstream of the GLM-GATA box. Moreover, in the two most highly expressed genes, a G-box-related motif and a CAAT motif were located a few bases upstream of the GLM-GATA box and the RY repeat motif, respectively. This may enhance the activator effect of CCRM4, which contains two additional motifs.

Our results also demonstrate significant differences in the expression levels in relation to the haplotypes of the promoters for *Glu-A1-1*, *-B1-1*, and *-B1-2*. For *Glu-A1-1*, the transcription from haplotypes h2 and h5 was severely reduced for the null allele. This is in agreement with previous data on SSP synthesis in developing grains of cv. Hereward, which also has a null allele (Shewry et al., 2009). A C/T change in the coding sequence of this null allele creates a premature stop codon that could explain why this gene is inactive (De Bustos et al., 2000). However, this does not explain the low levels of expression of these haplotypes as the qRT-PCR primers used to detect transcripts in this analysis are located upstream of this mutation. The very low transcription level of this null allele may be due to sequence polymorphism in the promoter as it has been demonstrated for the null *Glu-A1-2* allele (Halford et al., 1989). There were no obvious differences in our annotation of haplotypes of the *Glu-A1-1* promoter that could explain the large differences in expression observed. This is unlike the case of *Glu-A1-2*, which is silent and shows a particular *cis*-motif organization upstream of position −370 when compared with other y-type HMW-GS genes. However, a 277-bp fragment immediately upstream of the *GluA1-2* TSS was not able to generate any transcriptional activity (Halford et al., 1989). The organization of this fragment is quite similar to that of other expressed y-type promoters, so it is difficult to hypothesize how the gene is silenced. As expected, *Glu-B1-1* in Glenlea (line accession no. 3358) strongly expresses the Bx7 subunit transcript. This over-expression is explained by a 10.3-kb duplication including a second copy of *Glu-B1-1* (Ragupathy et al., 2008). Again, our annotation of the promoter alone does not show obvious differences that could explain the different levels of expression. In agreement with the results of Halford et al. (1989), the deletion found in the h3 haplotype does not impact the level of expression, which confirms that it plays no role in transcriptional regulation.

These results suggest that other mechanisms are able to modulate HMW-GS gene expression, such as *cis*-elements located further upstream of the region studied here. This would agree with results of Wang et al. (2013), who described the presence of key regulatory sequences in the distal sequence of *Glu-B1-1*, especially a Py-rich stretch at about position -2000. This sequence has been reported to cause a high level of expression in tomato (Daraselia et al., 1996). Methylation of DNA may also be involved in HMW-GS expression regulation, as shown for hordein genes in barley (Sorensen et al., 1996; Radchuk et al., 2005), even though no CpG islands were detected in the wheat promoter regions studied here using the PlantPAN search engine (Chang et al., 2008).

In conclusion, this work reveals a minimal regulatory framework shared by all the wheat HMW-GS gene promoters. The *cis*-elements organization is conserved, including all the motifs known to be involved in the regulation of SSP genes. The conservation of this regulatory framework strongly suggests that it is involved in the regulation of this gene family. The bipartite endosperm box was not found but a CCRM with the GATA-GLM box with an RY repeat and an AACA motif is present in all the promoters. The CCRMs, which occur at similar relative positions in all the promoters of this small family, presumably have a common evolutionary origin, suggesting that they may be functional. However, validating their functional roles requires further experiments. The “*in silico* footprint” described here will help to select motifs for functional validation, as shown here by transient expression assays of *Glu-B1-1* promoter. Our annotations do not directly account for differences in expression among promoter haplotypes, suggesting that other mechanisms may be involved in regulating HMW-GS gene expression.

### Conflict of interest statement

The authors declare that the research was conducted in the absence of any commercial or financial relationships that could be construed as a potential conflict of interest.
