# Supplementary material for: Conserved cis-regulatory modules in promoters of genes encoding wheat high-molecular-weight glutenin subunits
Source: Front Plant Sci. 2014 Nov 12;5:621. doi: 10.3389/fpls.2014.00621 (PMC4228979; doi:10.3389/fpls.2014.00621)
Supplement: Supplementary file 1 [file DataSheet1.PDF]

Supplementary Materials

Conserved *cis*-regulatory modules in promoters of genes encoding wheat high molecular weight glutenin subunits

Catherine Ravel[1,2*], Samuel Fiquet[1,2], Julie Boudet[1,2], Mireille Dardevet[1,2], Jonathan Vincent[1,2], Merlino[1,2], Robin Michard[1,2], Pierre Martre[1,2]

[1] INRA, UMR1095, Genetics, Diversity and Ecophysiology of Cereals, 5 chemin de Beaulieu, F-63 100 Clermont-Ferrand, France
[2] Blaise Pascal University, UMR1095, Genetics, Diversity and Ecophysiology of Cereals, F-63 177Aubière, France

*** Correspondence:** Catherine Ravel, INRA, UMR1095, Genetics, Diversity and Ecophysiology of Cereals, 5 chemin de Beaulieu, F-63 100 Clermont-Ferrand, France.
catherine.ravel@clermont.inra.fr

1. Supplementary Figures and Tables

1.1. Supplementary Tables

Supplementary Table 1 | Primers and PCR conditions for amplification of the HMW-GS gene promoters.

	Primer sequence		PCR conditions		
Gene	Forward (5'-3')	Reverse (5'-3')	Annealing temperature (°C)[a]	Elongation time (min)	Fragment size (bp)[b]
Glu-A1-1	GAAGTGTATCGTCTACGGAGGC	GACTACCGCCGCAAAAAGA	65-55 (40)	1	~ 875
Glu-B1-1	CCTATGTTAATTTTAGACATGACTGG	TACTGCCGCAAAGAGGACCAGG	70-60 (40)	1	~ 770
Glu-B1-2	AGCTACCTTCCATTAGTCGG	GAAGATGTTCCCCAAAATATTAC	60 (40)	1	~ 819/ ~ -480
Glu-D1-1	GTTTGGCTAGTTCATTTGTCGTGA	CACTGTAGTTGCTCAGAGGCCT	65-55 (40)	1	~ 662/ ~ -633
Glu-D1-2	ACTGCCGCAAAGAGGACCAG	TGCAACCATGCATCAAAATTTC	70-60 (40)	1	~ 1138

[a] Interval of temperatures used for touch-down amplification program or the annealing temperature followed by the total number of PCR cycles in brackets.

[b] Fragment size followed by the number of nucleotides upstream of the start codon when the reverse primer hybridized downstream of the start codon.

Supplementary Table 2 | List of primers used in this study for gene expression analysis.

Gene	Primer sequence Forward (5’-3’)	Reverse (5’-3’)	Fragment size (bp)	Efficiency %
Glu-A1-1	CATGCCGACAGGTCGTAG	CTGTTGCGGAGAAGTTACACTTA	230	88
Glu-B1-1	GGTGCCGCCCCATCAC	GCAGGTATTCCCCAAAATATCAT	142	87
Glu-B1-2	CCACAAAATAGAGATCAATTCACTA	CACGAGGGTGATGACTACTGT	86	87
Glu-D1-1	AGCGGTTAGTCCTCTTTGTGG	CGGAGCTGCTGGTCCATG	157	90
Glu-D1.2	GTTAGCGCAGAGCAGCAAG	CCCTCCATCCGACACACTG	89	93
β-tubulin	CCATCAGTTGGTTGAGAATGC	CAAAGCTGGGAGTGGTCA	101	96
18S RNA	CCATCCCTCCTCCGTAGTTAGCTTCT	CCTGTCGGCCAAGGCTATATAC	151	93
GAPDH	TTCAACATCATTCCAAGCAGC	CGTAACCCAAAATGCCCTTG	220	92
eF1a	CAGATTGGCAACGGCTACG	CGGACAGCAAAACGACCAAG	227	99

Supplementary Table 3 | DNA oligonucleotides used in EMSA.

Motif name[a]	Sequence (5'-3')[b]
GLM1	atagatgt**TGTGAGTCA**ttggatag
glm1	atagatgt**TtTtAtTa**A̲ttggatag
GLM2	atagatat**TGTGAGTCA**gcatggat
glm2	atagatat**TtTtAtTaA**gcatgga
G-box	gccca**TTACGTGG**ctttagcagacc
G-box	gccca**TTctcTGG**ctttagcagacc

[a] The names of wild-type and mutated motifs are indicated in upper cases and italics, respectively.

[b] Bold and uppercase residues correspond to the sequences of the *cis*-motifs GLM1, GLM2 and G-box. Bold and lower case residues indicate mutations in these motifs.

Supplementary Table 4 | DNA accession numbers of HMW-GS gene promoters.

Gene	Promoter haplotype[a]	Accession line No.	GenBank accession No.
Glu-A1-1	h1	748	KM116475
	h2	2135	KM116478
	h3	2358	KM116476
	h4	4482	KM116477
	h5	8048	KM116480
	h6	13812	KM116479
Glu-B1-1[c]	h1	2135	KM116484
	h2	964	KM116481
	h3	4901	KM116483
	h4	6529	KM116485
	h5	13310	KM116482
Glu-D1-1[c]	h1	748	KM116493
	h2	2135	KM116495
	h3	6086	KM116496
	h4	13812	KM116497
	h5	15658	KM116494
Glu-B1-2	h1	2135	KM116486
	h2	964	KM116487
	h3	8048	KM116488
	h4	5399	KM116489
	h5	13310	KM116490
Glu-D1-2	h1	2135	KM116491
	h2	6086	KM1164927

[a] To avoid redundancy, only one sequence per haplotype (see **Table 1**) was submitted.
